# Early assessment of the pharmacokinetic and pharmacodynamic effects following acetylsalicylic acid loading: toward a definition for acute therapeutic response

**DOI:** 10.1007/s11239-023-02914-7

**Published:** 2023-12-08

**Authors:** Paul A. Gurbel, Kevin P. Bliden, Parshotam Kundan, Danielle Kraft, Rueshil Parekh, Sahib Singh, Aravind D. Babu, Anika P. Shah, Rahul Chaudhary, Udaya S. Tantry

**Affiliations:** 1 Sinai Center for Thrombosis Research and Drug Development, Sinai Hospital of Baltimore, Baltimore, MD 21209, USA; 2 Heart and Vascular Institute, University of Pittsburgh Medical Center, Pittsburgh, PA, USA; 3 Artificial Intelligence for Holistic Evaluation and Advancement of Cardiovascular Thrombosis, Pittsburgh, PA, USA

**Keywords:** Acetylsalicylic acid, Aggregation, Platelet, Pharmacodynamics, Pharmacokinetics, Thromboxane

## Abstract

Despite decades of investigations, the optimal assessment of the “therapeutic response” to early after loading dose of acetylsalicylic acid (ASA) remains unclear. Limited information is available on the relation between pharmacodynamic (PD) and pharmacokinetic (PK) measurements assessed immediately after ASA administration. Serial PD and PK analyses were performed immediately after a single 162 or 650 mg dose of chewed and swallowed ASA in ten healthy adults. ASA response was defined as > 95% inhibition of serum thromboxane (Tx)B_2_ < 550 aspirin reaction units (ARU) by VerifyNow Aspirin (VN) test, and ≤ 20% arachidonic acid (AA)-induced platelet aggregation (PA). Correlation analyses between PK and PD measurements and receiver operating characteristic (ROC) curve analyses were performed. ASA response measured by VN test and AA-induced PA was achieved within 30 min of ASA administration. A correlation was observed between ARU and AA-induced maximum PA (r = 0.69, p < 0.001), serum TxB_2_ (r = 0.74 and p < 0.001), and serum TxB_2_ inhibition (r = 0.79, p < 0.001). In ROC curve analyses, ≤ 558 ARU and ≤ 7% AA-induced PA were associated with > 95% inhibition of TxB_2_. 686 ng/ml plasma ASA cut-off point was associated with > 95% inhibition of serum TxB_2_, ≤ 7% 1 mM AA-induced PA, and ≤ 585 ARU. A modest ~ 50% inhibition of TxB_2_ inhibition was associated with marked inhibition of 1 mM AA-induced platelet aggregation by LTA. Our analyses demonstrated important relationships between pharmacodynamic, and pharmacokinetic parameters measured immediately following oral ASA and cutoff values for ARU and AA-induced PA that is associated with > 95% inhibition of serum TxB_2_.

## Introduction

Life-long acetylsalicylic acid (ASA) is the bedrock antiplatelet therapy in patients with established cardiovascular disease (CVD) to prevent recurrent thrombotic events. The antiplatelet effect is mainly attributed to the inhibition of platelet cyclooxygenase (COX)-1 activity and the subsequent inhibition of thromboxane (Tx) A_2_ generation and platelet aggregation (PA). The laboratory assessment of the antiplatelet effects of ASA to ensure antithrombotic efficacy involves measuring: (a) stable metabolites of TxA_2_ that reflect ASA-induced platelet inhibition in vivo. These include urinary 11-dehydro-TxB_2_ and serum TxB_2_. Urinary 11-dhydro-TxB_2_ has been proposed as a marker of total body COX activity; (b) *ex vivo* COX-1 dependent PA assays, specifically arachidonic acid (AA)-induced PA assays, and (c) pharmacokinetic (PK) measurements of ASA and salicylic acid in plasma. Pharmacodynamic (PD) assays are extensively used in drug development and in clinical and translational research [1, 2]. It was proposed and advocated by the FDA that > 95% inhibition of serum TxB_2_ is needed for ASA to impart clinical efficacy [3, 4]. The latter stringent definition was largely based on investigations conducted decades ago demonstrating that this cut point was associated with marked inhibition of total body TxA_2_ production as measured by stable urinary metabolite levels [5].

It has been demonstrated that inhibition of platelet function by acetylation of the COX-1 enzyme occurs within minutes after the administration of ASA [6]. However, despite decades of investigations, the relationship between PD measurements, such as inhibition of serum TxB_2_ and AA-indued PA by light transmittance aggregometry (LTA), and PK assessed immediately after ASA administration, remain unclear [7]. The latter associations have become more important in recent years following the development of new ASA formulations that strive to achieve faster and greater inhibition of platelet COX-1 activity [8]. Therefore, it is important to establish an optimal definition of an “adequate therapeutic response” to ASA based on PD measurements. The latter would also require translational research to link the method to the occurrence of thrombotic events.

The aim of the current study was to define the PD and PK measured immediately (up to 1 h) after 162 and 650 mg doses of chewed and swallowed ASA in healthy subjects. We used the VerifyNow (VN) Aspirin test; 1 mM AA-induced PA by LTA, and serum TxB_2_ to assess PD effects and measured plasma ASA and salicylic acid (SA) to determine pharmacokinetic (PK) effects. The second aim was to determine the relationship between PD and PK measurements. Frequent sampling immediately after ASA administration allowed the opportunity to carefully analyze these relationships and to further define the platelet response to ASA.

## Methods

### Study design

This open-label, single-dose, two-treatment method comparison study was conducted in 10 healthy volunteers who abstained from ASA use within 10 days of enrollment and NSAID use within 5 days of enrollment. The study was performed in accordance with the Code of Ethics of the World Medical Association (Declaration of Helsinki) and approved by the local institutional review board. All subjects provided informed consent. After the screening, subjects were randomly assigned to receive a single dose of 162 mg or 650 mg soluble ASA (Bayer Corporation, Whippany, NJ, USA). In our study, 162 mg ASA was included because the latter dose was indicated in the United States Food and Drug Administration approved labeling and also in the 2021 American Heart Association Guideline for Coronary Artery Revascularization [9, 10]. We also included a 650 mg dose to obtain variable levels of PD and PK values after immediate oral ASA administration. Subjects were asked to chew the tablets and then swallow them with approximately 240 ml of water. Venous blood samples were collected using an indwelling intravenous catheter prior to ASA administration (baseline) and at 2, 5, 10, 20, 30, and 60 min after administration of ASA for PD and PK measurements.

The VN test is a turbidimetric assay where a change in optical signal is measured following the binding of activated platelets to fibrinogen-coated beads in the presence of an agonist (Werfen, Bedford, MA, USA). The VN Aspirin test is a qualitative test where AA is used to activate platelets within the test cartridge, and the results are expressed as Aspirin Reaction Units (ARU). A predefined cutoff of < 550 ARU has been used to define ASA therapeutic response and ASA onset [4].

In the light transmission aggregometry (LTA) method, a change in light transmission is measured in platelet rich plasma following platelet aggregation induced by an agonist. In the current study, platelet aggregation measurements were carried out using a Chronolog Lumi-Aggregometer 490 4 + 4 with the AggroLink software package (Chronolog Corp. Havertown, PA, USA). Percent change in maximum platelet aggregation from baseline in response to 1 mM AA in platelet rich plasma was measured. A predefined cutoff of AA-induced platelet aggregation ≤ 20% was used to define therapeutic aspirin response and ASA onset [11].

For serum TxB_2_ measurements, blood samples were collected in glass tubes (BD, Franklin Lakes, NJ, Ref 366430) without any anticoagulant and incubated immediately in a 37 °C water bath for 60 min. Serum was separated after incubation and stored immediately at − 80 °C. For PK measurements of ASA and salicylic acid (SA), blood was collected in K_2_EDTA tubes (BD, Franklin Lakes, NJ, Ref 367856), processed immediately and plasma samples were stored at − 80 °C. Both serum and plasma samples were shipped on dry ice (− 80 °C) to Syneos Health Laboratories (Québec, Canada) for further analysis. Serum TxB2 was measured by a validated HPLC assay at Syneos Health Laboratories using ACE Excel 2 C18^®^ column (Advanced Chromatography Technologies LTD, Aberdeen, Scotland, United Kingdom) followed by tandem mass spectrometry (Sciex Canada, Concord, Ontario, Canada). The limits of quantification for TxB_2_ were 0.25 to 500 ng/ml.

Plasma concentrations of ASA and SA were also measured by HPLC/MS at Syneos Health Laboratories. The method included liquid/liquid extraction using diethyl ether under acidic conditions, followed by reverse-phase chromatography on an Aqua C18^®^ column (Phenomenex, Aschaffenburg, Germany). The limits of quantification for ASA and SA in plasma were 2.5 to 2500 ng/ml and 25 to 10,000 ng/ml, respectively. Both serum TxB_2_ and PK parameters were calculated by model independent (compartment-free) methods using WinNonlin^®^ software, version 4.1.a. (Pharsight Corporation, Mountain View, CA, USA).

Continuous variables were expressed as mean ± SD, and categorical variables were expressed as numbers and percentages. The change in aggregation from baseline was assessed using a paired t-test; if the normality assumption was not met, a non-parametric (Wilcoxon signed-rank) method was used. The dose groups were compared at each time point using an independent t-test. In response to observed non-linearity in the relationships, both linear and 2nd-degree polynomial regression analyses were conducted to assess the correlation between PD and PK measurements. Polynomial regression consistently provided a superior fit across the examined relationships and was thus used for further interpretation. Receiver operating characteristic (ROC) curve analysis was performed to assess the relation between PD and PK parameters. p < 0.05 is considered statistically significant.

## Results

The mean age was 31 years, and all subjects had vital signs and hematologic measurements within normal limits.

### Pharmacodynamics

**Fig. 1:**
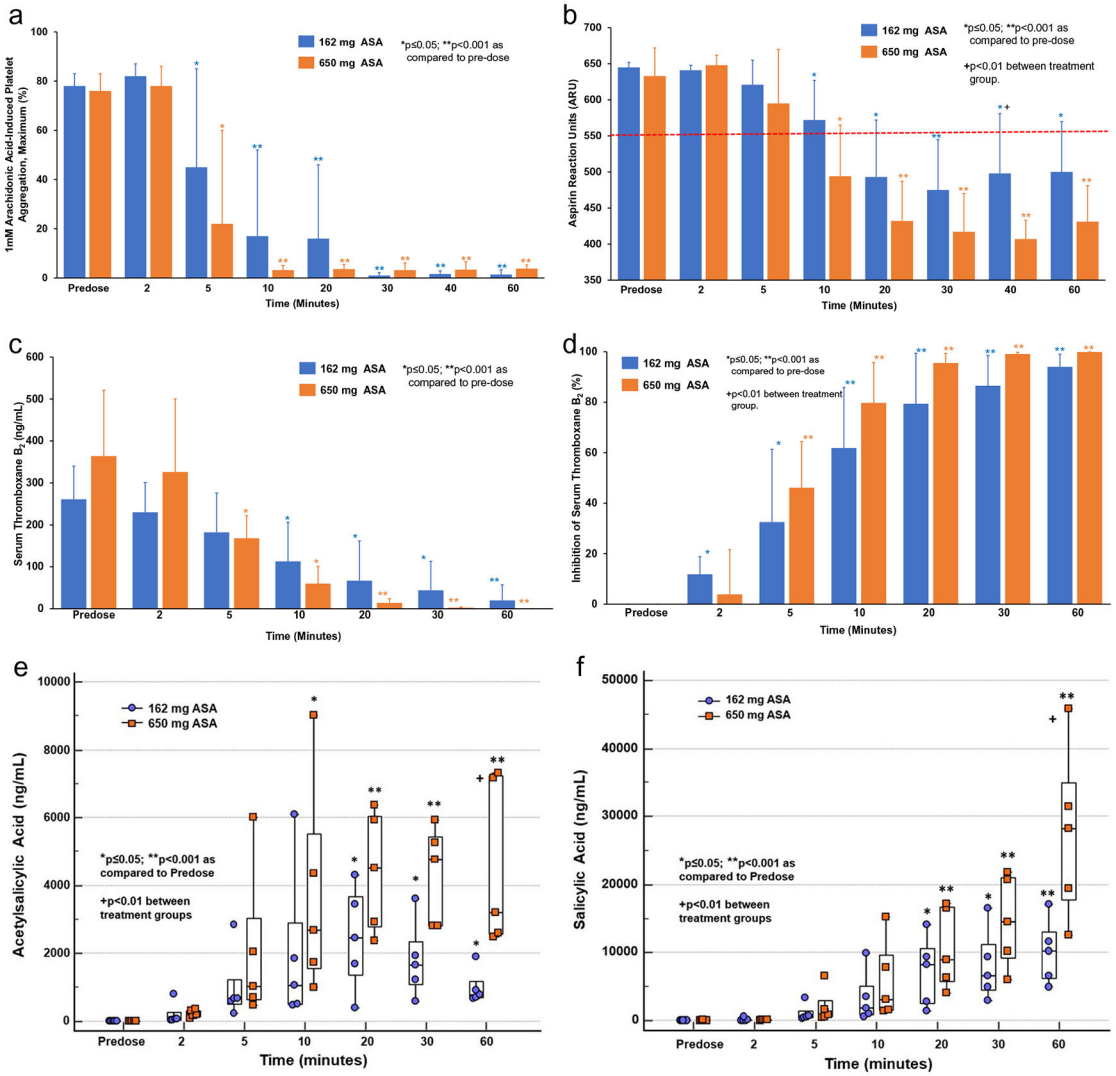
**a** 1 mM Maximum AA-Induced Platelet Aggregation. **b** Aspirin Reaction Units. **c** Serum Thromboxane B_2_. **d** Inhibition of Serum Thromboxane B_2_. **e** Plasma Acetylsalicylic Acid. **f** Plasma Salicylic Acid

Mean 1 mM AA-induced maximum PA was ~ 76% by LTA at pre-dose, and it reached a 20% PA threshold at 11 ± 11 min with 162 mg and 7 ± 3 min with 650 mg ASA (p = NS) (Fig. 1a). With VN Aspirin test, the mean ARU levels were > 630 at pre-dose in both groups indicating that all subjects were ASA naïve prior to dosing, which reached below the 550 threshold by 20 ± 7 min with 162 mg and 13 ± 7 min with 650 mg ASA (p = 0.07) (Fig. 1b).

Pre-dose TxB_2_ levels were 350 and 261 ng/ml and reduced to 1.1 and 0.4 ng/ml at 60 min with 162 and 650 mg ASA, respectively (p < 0.001). 95% inhibition of serum TxB_2_ level (therapeutic ASA response) was achieved at 38 ± 22 min and 22 ± 8 min with the 162 and 650 mg ASA doses, respectively (p = NS) (Fig. 1c, d).

### Pharmacokinetics

There was a gradual increase in plasma exposure to ASA and SA after ASA administration reaching maximum plasma ASA and SA levels of ~ 10,000 and ~ 27,000 ng/ml within 60 min with 162 mg and 650 mg ASA, respectively (Fig. 1e, f).

### Correlation between pharmacodynamic measurements

**Fig. 2:**
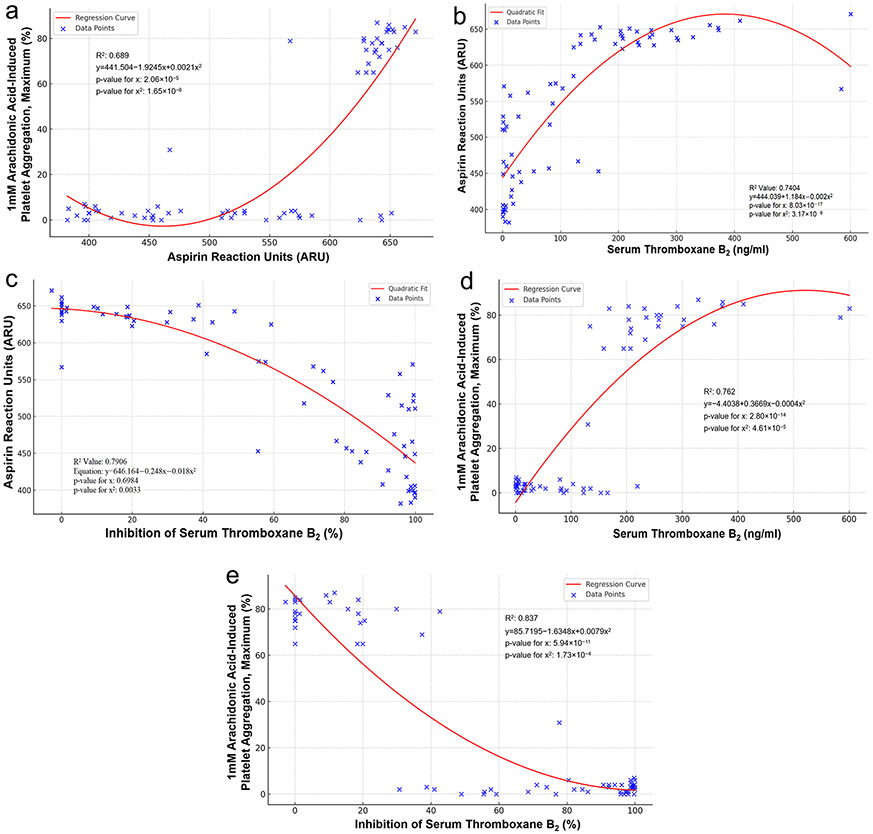
**a** Correlation between Aspirin Reaction Units and Arachidonic Acid-induced Platelet Aggregation. **b** Correlation between Aspirin Reaction Units and Serum Thromboxane B_2_. **c** Correlation between Aspirin Reaction Units and Inhibition of Serum Thromboxane B_2_. **d** Correlation between Arachidonic Acid-induced PA and Serum Thromboxane B_2_. **e** Correlation between Arachidonic Acid-induced PA and Inhibition of Serum Thromboxane B_2_

A good correlation was observed between VN ARU and 1 mM AA-induced maximum PA (r = 0.69, p < 0.001), serum TxB_2_ levels (r = 0.74, p < 0.001) and inhibition of serum TxB_2_ (r = 0.79, p < 0.001) (Fig. 2a-c). A strong correlation was observed between 1 mM AA-induced PA and serum TxB_2_ levels (r = 0.82, p < 0.001) and inhibition of serum TxB_2_ (r = 0.90, p < 0.001 (Fig. 2d, e).

Receiver Operator Characteristic (ROC) curve analysis indicated that ARU 550 was associated with ≥ 81 ng/ml serum TxB_2_ with an AUC 0.940 (specificity = 89.66, sensitivity = 90.91, p < 0.001) and a cut point of ≤ 558 ARU was associated with > 95% inhibition of serum TxB_2_ with an AUC = 0.912 (specificity = 74.47, sensitivity = 95.65, p < 0.001). ROC curve analysis indicated that a cut point of ≤ 7% maximum 1mM AA-induced PA was associated with > 95% inhibition of serum TxB_2_ with an AUC 0.728 (specificity = 54.35, sensitivity = 100, p < 0.001).

### Correlation between pharmacodynamic and pharmacokinetic measurements

The correlation between PK and PD measurements is shown in the Supplementary Figures 1a-d). Regression analyses demonstrated a correlation between ASA levels and serum TxB_2_ (r = 0.59, p < 0.001), inhibition of serum TxB2 (r = 0.75, p < 0.001) and ARU (r = 0.68, p < 0.001). The cutoff value of 686 ng/ml of ASA was correlated well with 1 mM-AA induced PA and ARU levels.

ROC curve analysis indicated that a cut point of > 686 ng/ml ASA was associated with > 95% inhibition of serum TxB_2_ (change from pre-dose) with an AUC 0.887 (specificity = 64.58%, sensitivity = 100%, p < 0.001) and a cut point of > 4907 ng/ml SA was associated with > 95% inhibition of serum TxB_2_ with an AUC 0.976 (specificity = 89.58%, sensitivity = 100%, p < 0.001).

In addition, the 686 ng/ml ASA cut off point was associated with ≤ 7% 1 mM AA-induced PA by LTA (AUC = 0.773, specificity = 76.19 and sensitivity = 95.00%, p = 0.001) and ≤ 585 ARU (AUC = 0.936, specificity = 90.48%, sensitivity = 92.68%, p < 0.001). Similarly, the 4907 ng/ ml SA cut off point was associated with ≤ 7% 1 mM AA-induced PA by LTA (AUC = 0.716, specificity = 54.55 and sensitivity = 100%, p = 0.0015) and ≤ 558 ARU (AUC = 0.955, specificity = 81.82%, sensitivity = 96.55%, p < 0.001).

## Discussion

Based on the analysis of PD and PK during ASA onset, we demonstrated that (a) the immediate PD response to ASA is well characterized by COX-1 specific assays; AA-induced PA by LTA, VN Aspirin test and serum TxB_2_ measurements; (b) a stepwise relation exists between ASA response assessed by COX-1 specific platelet function assays and the gold standard of > 95% inhibition of serum TxB_2_; and (c) specific PK cutoffs, specifically ASA levels are associated with the onset of high platelet inhibition. This is the first study to evaluate the onset of PD effects induced by ASA by the VN Aspirin test, demonstrating that this method should be suitable for translational research investigations as it correlates well with the results of conventional aggregometry.

Many factors associated with ASA pharmacology are relevant to its clinical efficacy. However, the level of serum TxB_2_ or its inhibition, the level of urinary 11-dehydro TxB_2_ production, or the degree of PA have not been precisely established for determining adequate cardioprotection by ASA. An association between PD measurements and thrombotic clinical occurrences have been reported. In retrospective analyses from two large-scale clinical trials, patients with the upper quartile of 11-dehydro TxB_2_ levels had 1.66–1.8 times-higher risk for myocardial infarction (MI), stroke, or CV death than those in the lower quartile [12, 13]. In another study, patients undergoing PCI who had a serum TxB_2_ > 3.1 ng/ml measured after at least 3 days of 81 or 325 mg daily ASA had a worse two-year occurrence of CV death, MI, hospitalization for revascularization, or acute coronary syndromes (28.9% in > 3.1 ng/ml TxB_2_ vs. 19.0% in serum TxB_2_ ≤ 3.1 ng/ml) [14]. However, other studies failed to establish a link between PD effects of ASA and thrombotic risk. For example, in the large ADAPT DES PCI registry (n > 8000), > 550 ARU was not associated with oneyear ischemic events but was inversely associated with less bleeding (adjusted HR 0.65) [15]. In the Antiplatelet Drug Resistances and Ischemic Events (ADRIE) study of 771 patients with stable CVD who were on ASA, clopidogrel, or both, patients with ≥ 12 ng/ml TxB_2_ (poor responders to ASA) did not have greater thrombotic risk [16]. Similarly, in another study of 900 patients with stable coronary artery disease, 3-year CV events in the highest quartile measured by the VN ASA test had similar thrombotic outcomes to those in the lowest quartile [17]. Taken together, these results suggest that except for urinary 11-dehydro TxB_2_, ASA response measured by other laboratory methods has not been firmly linked to thrombotic risk. These translational research studies assessed ASA response using a single measurement and a single test during a steady state of ASA administration. Although serial PD measurements have been undertaken in earlier studies of ASA response, an extensive analysis of the relation between specific PD assays and PK measurements has not been explored. In addition, some had reported a poor correlation between the results of different PD assays when ASA response was measured only once [18].

In the current study, we assessed the immediate response to ASA administration using serial PD and PK measurements. Our data presented new information on the correlation between different PD assays at this period. We have demonstrated that the onset of detectable antiplatelet effects of ASA occurs within a few minutes of a single oral administration. The latter observation suggested that the correlation between assays is greater using serial earlier time points, unlike previous studies.

Ischemic events occur in patients with arterial disease following the occlusion of arteries at the site of vascular injury. This process is critically dependent on the formation of stable platelet aggregates; adenosine diphosphate (ADP) and TxA_2_ play key roles in the amplification of platelet activation that drives platelet function to occlude the lumen at the site of such vascular injury. Therefore, an analysis of PA (function) has been proposed as a better indicator of thrombotic risk than the measurement of a soluble marker of platelet activation [19]. The current study results provide evidence that platelet aggregation measurements correlate with TxB_2_ levels and inhibition of TxB_2_. This relationship appears to be dichotomous. Only modest inhibition of TxB_2_ (~ 50% inhibition) was associated with marked inhibition of 1 mm-AA induced platelet aggregation by LTA. In the ROC curve analyses, the cutoff values for “adequate” ASA response indicated by ≤ 7% 1 mM AA-induced PA by LTA and ≤ 558 ARU by VN ASA test were highly sensitive for > 95% inhibition of TxB_2_. It is interesting again to note that these cutoff values are similar to the suggested “therapeutic” cutoff values for ASA response by LTA (< 20% AA-induced PA) and < 550 ARU by VN test reported in the literature [1, 11, 20].

With respect to PK measurements, the sequential decrease in platelet function in PD tests was reflected in the sequential increase in PK measurements. In the ROC curve analyses, both ASA and SA levels were associated with > 95% inhibition of TxB_2_ with cutoff values of > 686 ng/ml and > 4907 ng/ml, respectively, with high AUC and 100% sensitivity. The cutoff value of > 686 ng/ml ASA was associated with ≤ 7% 1 mM AA induced PA, ≤ 585 ARU, and > 95% inhibition of serum TxB2 in the regression analysis. Given the modest specificity of ≤ 7% 1 mM AA-induced PA and ≤ 585 ARU, the latter markers cannot serve as surrogates for > 95% inhibition of serum TxB_2_. Moreover, these novel observations indicate that 1 mM AA-induced PA with a cutoff value of ≤ 7% and VN Aspirin test with < 558 may be a surrogate for “adequate” inhibition by ASA. However, only translational research will prove the latter hypothesis. A correlation between LTA and VN Aspirin test results suggests that the latter point-of-care assay can be easily used in multicenter PD studies of ASA. We believe that a total of 560 PD and PK measurements in the current study is sufficient to reach our conclusions [21, 22]. The current PD and PK measurements were comparable to earlier studies, where these measurements were performed repeatedly within 1 h [8, 23]. We studied the systemic venous samples to analyze PK and PD tests. Since aspirin irreversibly inhibits platelet COX-1 enzyme when platelets are exposed to higher levels of ASA in the portal circulation, the PK and PD measurements done on peripheral venous samples may not represent the true drug concentration thresholds. However, it is not possible to collect portal circulation blood samples in human studies. Finally, the strength of our conclusions would have been enhanced by including a larger number of subjects.

In conclusion, our study extensively defined the PD and PK profiles immediately following ASA loading and demonstrated the relation between LTA and point-of-care test measurements, supporting the latter as a suitable method for future translational research. We demonstrated a dichotomous relation between TxB_2_ levels/inhibition and measurements of platelet function. Approximately 50% inhibition of TxB_2_ is required to reach high levels of platelet function inhibition. We established PK cut-off points of ASA and SA that may be useful in future translational investigations involving patients with cardiovascular disease who may have a higher baseline circulating level of thromboxane and higher platelet reactivity phenotype. These markers need to be studied in relation to clinical event occurrences in future studies.

## Supplementary Material

Supplement

## Highlights

- The optimal assessment of the “therapeutic response” to ASA is unclear.
- Serial PD and PK analyses were performed immediately after a single 162 or 650 mg dose of chewed and swallowed ASA in ten healthy adults.
- A correlation was observed between platelet function assessed by VerifyNow test and AA-induced platelet aggregation, serum thromboxane B_2_ levels and inhibition of thromboxane B_2_.
- A modest ~ 50% inhibition of TxB_2_ inhibition was associated with marked inhibition of 1 mm-AA induced platelet aggregation by LTA.
