# Supplementary figures and images for: Early assessment of the pharmacokinetic and pharmacodynamic effects following acetylsalicylic acid loading: toward a definition for acute therapeutic response
Source: J Thromb Thrombolysis. Author manuscript; Available in PMC 2025 Jan 1. (PMC10830588; doi:10.1007/s11239-023-02914-7)

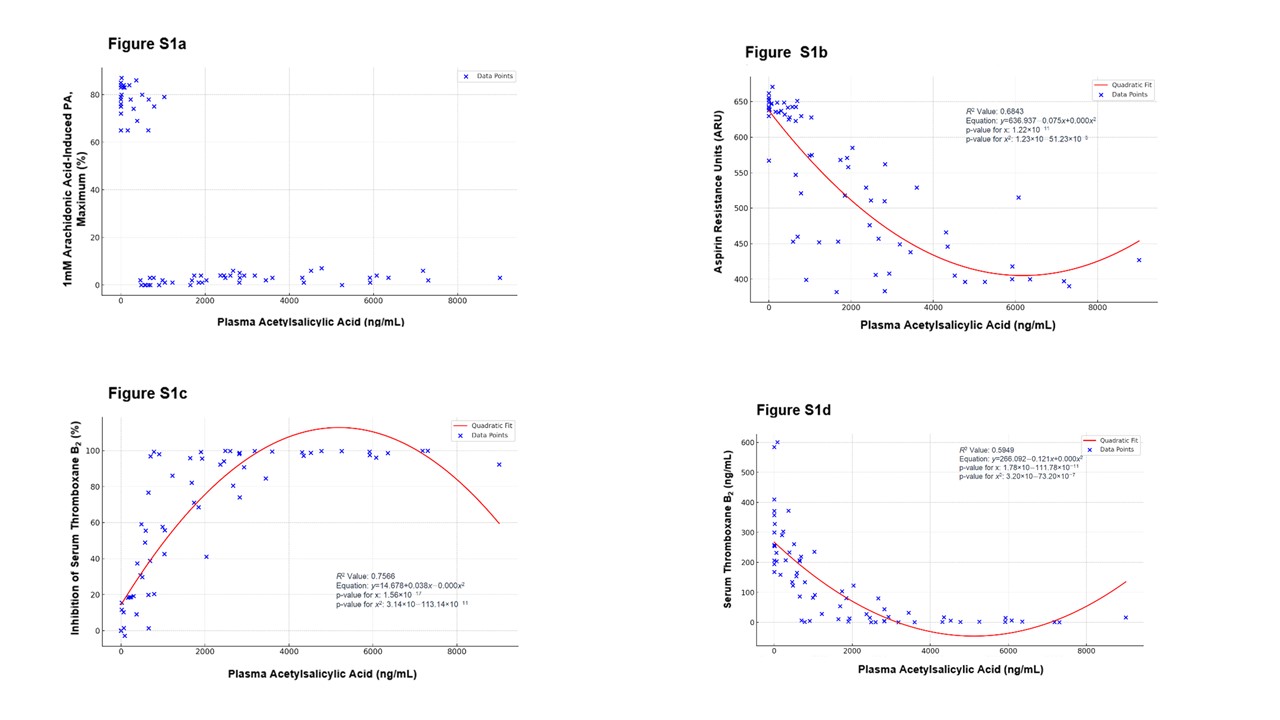

Supplement: Supplement [file NIHMS1959804-supplement-Supplement.jpg]
